# Literary and Journalistic Notes

**Published:** 1905-01

**Authors:** 


### LITERARY AND JOURNALISTIC NOTES.

THE *Annals of Surgery* for December, 1904, issues an anniversary number, commemorating the twentieth year of its publication, containing 282 pages, and is copiously illustrated. The contributors are Johannes Orth, J. William White, W. Watson Cheyne, J. Collins Warren, Frank W. Foxworthy, George Emerson Brewer, James H. Nicoll, Roberto Alessandri, Brennan Dybalt, William J. Mayo, James P. Warbasse, Charles L. Scudder, Francis J. Shepherd, Harry H. Germain, Harry Atwood Fowler, Walter B. Odiorne, Channing C. Simmons, and Francis S. Watson. It is edited by Lewis S. Pilcher, J. William White, Sir William Macewen, and W. Watson Cheyne, and is published by J. B. Lippincott Company, Philadelphia. It is the best periodical exponent of general surgery published in the English language.

---

THE *American Journal of Nursing,* which is in the fifth year of its publication, announces that it stands at the head of a movement for state registration and higher preliminary and technical education for nurses. It is published by J. B. Lippincott Company, Philadelphia.

MESSRS. LEA BROTHERS & COMPANY, of Philadelphia and New York, publish a general catalogue, with annual revision, of all medical, surgical, pharmaceutical, dental, and veterinary books in the English language. It is furnished gratis on application to the publishers.

